# Idiopathic Chylothorax During Pregnancy: A Case Report

**DOI:** 10.7759/cureus.47841

**Published:** 2023-10-27

**Authors:** Roberto de Jesús Martínez Camacho, Lilia Itzel Martínez Camacho, Daira Martínez Camacho, Ania Martínez Camacho

**Affiliations:** 1 Department of Internal Medicine, Hospital General de Zona (HGZ) 1, Instituto Mexicano del Seguro Social, Zacatecas, MEX; 2 Faculty of Medical and Biological Sciences “Dr. Ignacio Chávez”, Universidad Michoacana de San Nicolás de Hidalgo (UMSNH), Morelia, MEX; 3 Faculty of Medicine, Universidad Autónoma del Estado de México, Toluca, MEX

**Keywords:** pleural effusion, non traumatic, idiopathic, pregnancy, chylothorax

## Abstract

The diagnosis of chylothorax during pregnancy is rare and is generally triggered during labor. The study of pleural fluid and imaging studies can facilitate diagnosis, even though determining its etiology can be complicated. This case involves a young pregnant woman presenting with disabling dyspnea and chest pain due to pleural effusion. The conventional study of the pleural fluid was not conclusive; however, the measurement of chylomicrons guided us to determine the diagnosis of chylothorax. Based on imaging studies and specific markers, some of the main etiologies were not determined, leading to classification as idiopathic. After drainage and dietary adjustment, a significant improvement in symptoms was achieved with the help of an interdisciplinary team, which is crucial for the prevention of complications and adequate resolution of the pregnancy.

## Introduction

Chylothorax occurs secondary to the accumulation of lymph in the pleural space. It is classified as either traumatic or nontraumatic, with the former being more common. Among the nontraumatic causes, which are very rare, lymphoma and other malignancies, systemic diseases, and anatomical and obstructive malformations of lymph nodes predominate; approximately 5-10% are not identified and fall into the idiopathic category [1]. The mechanisms involved in its pathophysiology are the extravasation of lymph secondary to an interruption or obstruction that increases pressure in the lymphatic vessels. Other factors, such as increased pressure in pulmonary circulation on the right chambers of the heart, have an effect on the final pressure of the thoracic duct and can trigger it [2,3]. Most cases are unilateral, predominantly right, depending on the anatomical level where the leak occurs. The degree of injury and the patient’s diet determine the speed at which the fluid accumulates, which will lead to the appearance of symptoms [4]. Development of chylothorax during pregnancy is very rare; it is commonly associated with childbirth given the excessive increase in intrathoracic and abdominal pressure that can exacerbate the damage already established by other etiologies. Its diagnosis is achieved with the study of pleural fluid in which high levels of triglycerides and the presence of chylomicrons predominate.

## Case presentation

A 26-year-old gravida 3, para 2 with a gestational age of 21 weeks, presented to our emergency department with a two-week history of stabbing chest pain in the lower right thoracic region, which was associated with dyspnea and had progressively worsened to Grade 3 of the modified Medical Research Council (mMRC) dyspnea scale. She reported no food intake for the past two days, associated with mild nausea, and denied the presence of fever or history of upper respiratory tract infections. She denied a history of chronic degenerative disease and the use of any medications and reported being a nonsmoker. She had two prior pregnancies without complications.

**Figure 1:**
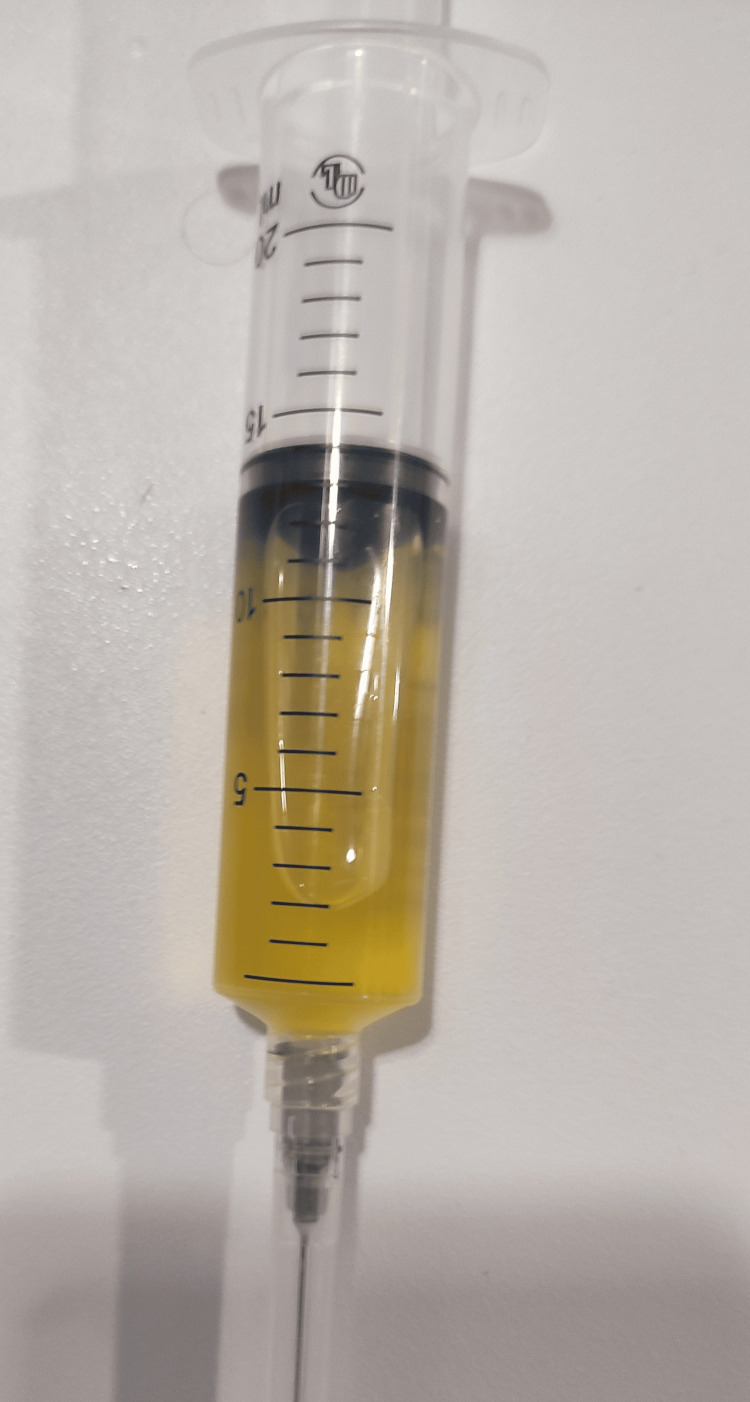
Turbid yellow pleural fluid immediately after thoracentesis

On arrival, her vital signs were within normal limits, except she had an oxygen saturation of 90% on room air. Upon physical examination, she had intact costal arches, decreased movements, and integration of pleural effusion syndrome in the right hemithorax. An ultrasound revealed the presence of abundant fluid with heterogeneous characteristics. A chest X-ray confirmed a unilateral pleural effusion involving approximately one-third of the pleural space, without other radiographic abnormalities of the heart or lungs. We performed an ultrasound-guided diagnostic thoracentesis that yielded 60 ml of pleural fluid (Figure 1); cytology reported post-centrifugation characteristics of yellow, milky fluid with a cholesterol level of 101.5 mg/dl and triglycerides of 68.5 mg/dl. According to Light’s criteria, it was an exudative effusion; however, it did not meet the criteria for chylothorax by triglyceride levels. Microbiology studies were negative. Because the result was inconclusive, we sent the pleural fluid for an assay of chylomicrons, which measured 185 mg/dl, confirming the diagnosis of chylothorax (Table 1). We requested an evaluation by the gynecology service, which determined the patient was experiencing a normal evolutionary pregnancy, and initiated further studies to determine the etiology.

**Table 1:** Laboratory Investigations and Result IgG: immunoglobulin G; IgM: immunoglobulin M

PLEURAL FLUID		
Investigation	Result	Reference range
Cell Count	4110 Al/mm^3^	1000-5000 Al/mm^3^
Polymorphonuclear	20%	<50%
Aspect	Milky	
Color	Yellow	
Coagubility	Absent	
Density	1.02	1.006-1.015
pH	8	7.3
Glucose	48 mg/dl	50-90 mg/dl
Proteins	4.65 g/dl	< 0.5 g/dl
LDH	910 u/l	< 200 u/l
Cholesterol	101.5 mg/dl	< 60 mg/dl
Amylase	61 u/l	
Triglycerides	63.5 mg/dl	
Chylomicrons	185 mg/dl	
BLOOD PANEL		
Investigation	Result	Reference range
Glucose	79.3 mg/dL	65-110 mg/dL
Urea Nitrogen	8.79 mg/dL	7-25 mg/dL
Creatinine	0.5 mg/dL	0.7-1.5 mg/dL
Sodium	135 mmol/L	137-145 mmol/L
Potassium	3.85 mmol/L	3.5-5.1 mmol/L
Chloride	100.9 mmol/L	98-107 mmol/L
Calcium	8 mg/dL	8.4-10.2 mg/dL
Total Protein	5.76 g/dL	<6.3 g/dL
Albumin	3.15	
Cholesterol	173	
Triglycerides	162.1	
Lactate Dehydrogenase	168 U/L	< 250 U/L
White Blood Cell Count	7.7 Thousand/uL	5.0-10.0 Thousand/uL
Red Blood Cell Count	Million/uL	4.20-5.80 Million/uL
Hemoglobin	11.9 g/dL	12-16 g/dL
Hematocrit	37.9 %	38-47%
Platelet Count	285 Thousand/uL	150-400 Thousand/uL
Absolute Neutrophils	6.09 Thousand /uL	1.5-5.0 Thousand /uL
Absolute Lymphocytes	0.66 Thousand /uL	0.5-1.5 Thousand /uL
Absolute Eosinophils	0.02 Thousand /uL	0-1 Thousand /uL
AUTOIMMUNE PANEL		
Investigation	Result	Reference range
Anti-cardiolipin Antibodies (IgG)	1.39 U GPL	Negative< 10
Anti-cardiolipin Antibodies (IgM)	9.77 U MPL	Negative< 10
Lupus Anticoagulant Test	26.1	Negative< 38 seg
Anti-Beta-2-Glycoproteins IgG	7.9 SG U/ml	Negative< 10
Anti-Beta-2-Glycoproteins IgM	8.4 SM U/ml	Negative< 12
Anti-Smith Antibodies	Negative	Negative
Anti-DNA Antibodies	Negative	Negative
Antinuclear Antibodies	Negative	Negative

**Figure 2:**
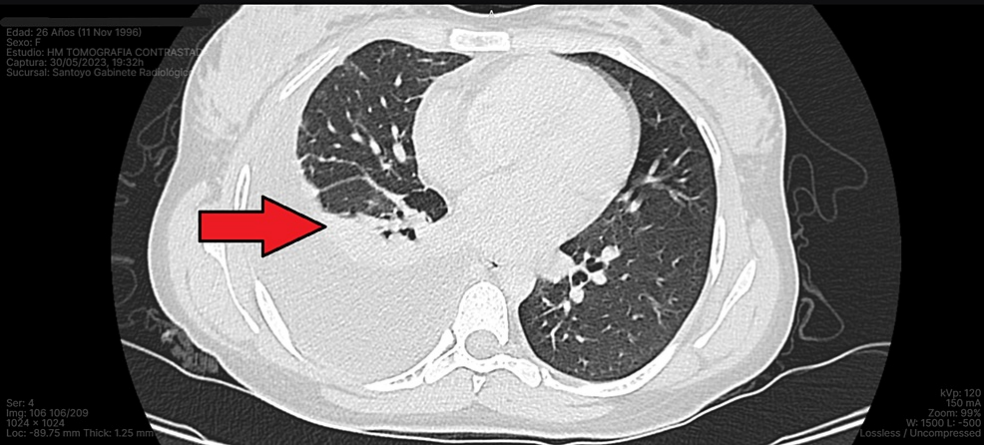
CT scan of the chest lung window showing right-sided pleural effusion

**Figure 3:**
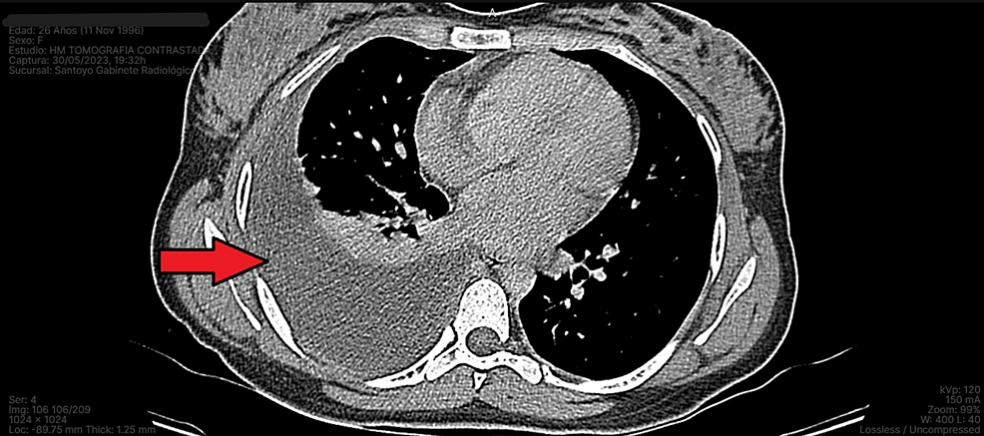
CT scan of the chest mediastinal window showing right-sided pleural effusion

Laboratory studies showed no abnormalities and pleural fluid culture and the viral panel were negative. Serial smears of acid-fast bacilli were performed and a sample was sent for nucleic acid amplification testing to rapidly detect mycobacterium; the results were negative. We obtained additional imaging studies, including a contrast-enhanced computed tomography of the chest and abdomen, which showed the pleural effusion was confined exclusively to the right chest (Figures 2, 3). There were no abnormal lymphatic or bronchial findings, no evidence of cysts or tumors, and the liver and spleen appeared to be normal. The electrocardiogram and echocardiogram did not show functional or anatomical alterations for the patient’s age. Rheumatology determined the chylothorax was not related to autoimmune causes in the absence of autoantibodies. After ruling out the most common etiologies, the chylothorax was classified as idiopathic.

Because of the severity of respiratory symptoms, we performed therapeutic thoracentesis, obtaining approximately 3 liters of chylous fluid. The remainder of her hospital course was uneventful. Because she was pregnant and her symptoms had subsided, we opted for conservative management, recommending a high-protein, low-fat diet. Because she did not experience further dyspnea or pain and a follow-up ultrasound revealed no increase in fluid, we discharged her from the hospital.

During the patient’s follow-up in the outpatient clinic, the patient remained symptom-free. An insignificant amount of pleural fluid was present and despite normal prenatal development, the pregnancy was classified as high risk, and the patient was referred to a specialized center for evaluation by gynecology, which planned surgical delivery to avoid the complications associated with childbirth.

## Discussion

The symptoms of dyspnea, pleuritic pain, and cough are common in chylothorax and are associated with up to 56% of cases. Chyloptysis is a rare symptom (1.2%), with reported cases having an absence of respiratory symptoms, related to the rate of liquid formation [1].

Although the characteristics of the fluid in our patient included a milky appearance after centrifugation, this is a characteristic that occurs in less than 50% of cases. Pleural fluid in chylothorax can be serous, bloody, yellow, or greenish and have an alkaline pH; however, acidosis is present in infectious processes. The cellularity has <20% polymorphonuclear cells, the laboratory did not report the lymphocyte values, in which the predominance in chylothorax is up to 80%. By modified light criteria, it met the characteristics of exudate, the most common pattern [5,6].

The diagnostic criteria are a triglyceride level greater than 110 mg/dL (1.24 mmol/L) and a cholesterol level less than 200 mg/dL (5.2 mmol/L). The ratio of pleural fluid to serum triglycerides should be >1 and the ratio of pleural fluid to serum cholesterol should be less than 1. The diagnosis is unlikely when the triglyceride level is <50 mg/dL (0. 56 mmol/L), having to rule out the presence of pseudochylothorax, which is characterized by a triglyceride level <50 mg/dL (<0.56 mmol/L) with a cholesterol level >200 mg/dL (> 5.18 mmol/L) [7]. Diagnosis can be complicated when triglyceride levels are within the range of 50 to 110 mg/dL. Nutritional status is directly related to the level of triglycerides in pleural fluid. Before taking the sample, our patient had been without adequate food intake for four days due to her symptoms. The presence of chylomicrons in the pleural fluid is pathognomonic of chylothorax and can be identified through the use of lipoprotein electrophoresis; it is the gold standard for diagnosis [8,9].

In the differential diagnosis, the most common cause of nontraumatic chylothorax is malignancy, with lymphoma representing more than 70% of cases and non-Hodgkin’s lymphoma being the most prevalent. Primary disorders, such as lymphangioleiomyomatosis, characterized by the proliferation of smooth muscle, affect women of childbearing age; pulmonary cysts are characteristic in imaging studies. Other less common causes are infections, congenital hemangiomatosis, yellow nail syndrome, systemic lupus erythematosus, Behçet’s disease, sarcoidosis, and amyloidosis. Tuberculous effusion is a well-described cause of pseudochylothorax or cholesterol effusion; diagnosis is established microbiologically with a positive pleural fluid or sputum, bronchoalveolar lavage, mycobacterial culture, or a positive nucleic acid amplification test. In 9% of cases, the cause remains unknown and is classified as nontraumatic idiopathic chylothorax [4,9].

Initial imaging studies, chest X-ray, and ultrasound can confirm the presence of pleural effusion, and initially detect lung malignancies, macroscopic hilar lymphadenopathy, or chest wall trauma. Even though computed tomography with or without contrast has low resolution for lymphatic circulation, we choose it because it can rule out lymphadenopathy and solid tumors, and study the mediastinal and retroperitoneal structures to limit the broader differential diagnosis in the context of chylothorax. In our case, being a pregnant woman, this study method was useful to rule out the main causes and not undergo an invasive test such as conventional lymphangiography or magnetic resonance imaging. This can provide therapeutic value by identifying the defect. However, these are studies that are difficult to access in our environment [7-9].

Definitive treatment addresses the underlying cause. In idiopathic chylothorax, the underlying cause is unknown by definition. Thus, in our case, we instituted a conservative treatment, which included therapeutic thoracentesis to relieve the symptoms, and exercised caution because excessive removal of pleural fluid can cause serious electrolyte alterations. Dietary measures included a diet high in protein with low-fat medium-chain triglycerides, which reduces the concentration at the level of the thoracic duct and reduces splenic flow and hepatic venous pressure (and therefore lymphatic flow), thus limiting the accumulation of pleural fluid. It is appropriate to use pharmacological therapy when chylothorax continues to recur despite appropriately established dietary measures. Somatostatin and its analogs are the most studied drugs that reduce gastrointestinal absorption and splenic flow. Generally, one or two weeks of therapy are required before assessing a response. The use of α1 adrenergic agonists, such as midodrine and etilefrine, remains limited, and studies evaluating these agents are lacking. When the daily chyle leak volume exceeds 1.0 to 1.5 L in a day for more than five to seven days, other therapies might be employed, such as chemical pleurodesis, pleuroperitoneal diversion, embolization of the thoracic duct (or even ligation), surgery of the thoracic duct through open or assisted thoracotomy, and closure of the defect. In the case of our patient, she had adequate improvement with conservative therapy [9,10].

## Conclusions

The presentation of chylothorax during pregnancy is rare and is mainly associated with labor due to increased intrathoracic pressure. In this case, the initial study of the pleural fluid did not meet all the criteria, and lipoprotein electrophoresis to identify chylomicrons was very important to reach the diagnosis. We were unable to identify a cause with imaging methods. The approach must be by a multidisciplinary team in order to identify the cause and, in the case of pregnancy, initiate treatment in order to avoid complications.
